# Steatotic liver disease and its newly proposed sub-classifications correlate with progression of the coronary artery calcium score

**DOI:** 10.1371/journal.pone.0301126

**Published:** 2024-03-26

**Authors:** Aryoung Kim, Danbee Kang, Sung Chul Choi, Juhee Cho, Dong Hyun Sinn, Geum-Youn Gwak

**Affiliations:** 1 Department of Medicine, Samsung Medical Center, Sungkyunkwan University School of Medicine, Seoul, South Korea; 2 Department of Internal Medicine, Inje University Ilsan Paik Hospital, Goyang, South Korea; 3 Department of Clinical Research Design and Evaluation, SAIHST, Sungkyunkwan University, Seoul, South Korea; 4 Center for Clinical Epidemiology, Samsung Medical Center, Sungkyunkwan University, Seoul, South Korea; 5 Center for Health Promotion, Samsung Medical Center, Sungkyunkwan University, Seoul, South Korea; 6 Departments of Epidemiology and Medicine and Welch Center for Prevention, Epidemiology and Clinical Research, Johns Hopkins Medical Institutions, Baltimore, MD, United States of America; Al-Azhar University, EGYPT

## Abstract

**Background & aims:**

A new nomenclature, Steatotic Liver Disease (SLD), has been proposed by consensus with sub-classifications and requires evidence-based validation. We assessed whether the presence and severity of SLD, as well as its sub-classifications, are associated with the progression of coronary atherosclerosis.

**Methods:**

This longitudinal cohort study included 13,811 adults who participated in repeated regular health screening examinations between January 1, 2004 and December 31, 2021 that included assessments of their coronary artery calcium (CAC) scores. SLD was defined using abdominal ultrasonography and classified as metabolic dysfunction associated steatotic liver disease (MASLD), MASLD with increased alcohol intake (MetALD), and cryptogenic SLD. SLD severity was assessed using fibrosis-4 (FIB-4) scores. The progression of CAC scores was measured using multidetector CT scans.

**Results:**

The average duration of follow-up was 5.8 years. During follow-up, the annual rate of CAC progression in participants with and without SLD was 18% (95% CI 17%–19%) and 14% (95% CI 13%–14%) (p < 0.01), respectively. The multivariable ratios of progression rates when we compared participants with cryptogenic SLD, MASLD, or MetALD with those without SLD were 0.98 (95% CI 0.95–1.01), 1.03 (95% CI 1.03–1.04), and 1.07 (95% CI 1.04–1.09), respectively. The multivariable ratios of progression rates when we compared participants with SLD with FIB-4 score <1.3 and SLD with FIB-4 score ≥1.3 with those without SLD were 1.03 (95% CI 1.02–1.04), and 1.05 (95% CI 1.04–1.06), respectively.

**Conclusions:**

SLD was associated with a higher risk of coronary atherosclerosis, and the risk differed by sub-classifications and severity. These findings suggest that the newly proposed definition has clinical relevance in terms of stratifying cardiovascular disease risk.

## Introduction

Nonalcoholic fatty liver disease (NAFLD) is a condition in which the liver accumulates fat without significant alcohol intake, viral hepatitis, medications that could cause fatty liver, or other obvious causes [1]. Although the NAFLD nomenclature has traditionally been widely used, it has always been understood that the term “non-alcoholic” does not accurately reflect the etiology of the disease, and the term “fat” has been regarded as stigmatizing for some people. Recently, a multi-society Delphi conference published a new term, Steatotic Liver Disease (SLD), to encompass the various etiologies of steatosis. The conference also proposed sub-classifications (cryptogenic SLD, metabolic dysfunction–associated steatotic liver disease (MASLD), and metabolic dysfunction-associated alcoholic liver disease [MetALD]) based on cardiometabolic risk factors and alcohol consumption [2]. It is imperative to validate the applicability of these new criteria in a real cohort to determine whether they accurately reflect the nature and prognosis of the disease.

One fatal outcome of human fatty liver disease is cardiovascular disease (CVD) [3]. Thus, the value of the newly proposed definition and sub-classifications for stratifying CVD risk warrants validation. Furthermore, one of the newly proposed sub-classifications, MetALD, has not been evaluated for its clinical significance. Coronary artery calcium (CAC) score is a reliable marker of subclinical atherosclerosis, and can independently predict the future CVD event risk [4, 5]. CAC progression is associated with the development of incident coronary heart disease and all-cause mortality, with an approximately linear dose-response relationship [6, 7]. In this study, we assessed the association between the newly defined SLD and each of its sub-classifications and CVD risk by analyzing CAC progression in a large sample of asymptomatic adults.

## Methods

### Study population

**Fig 1:**
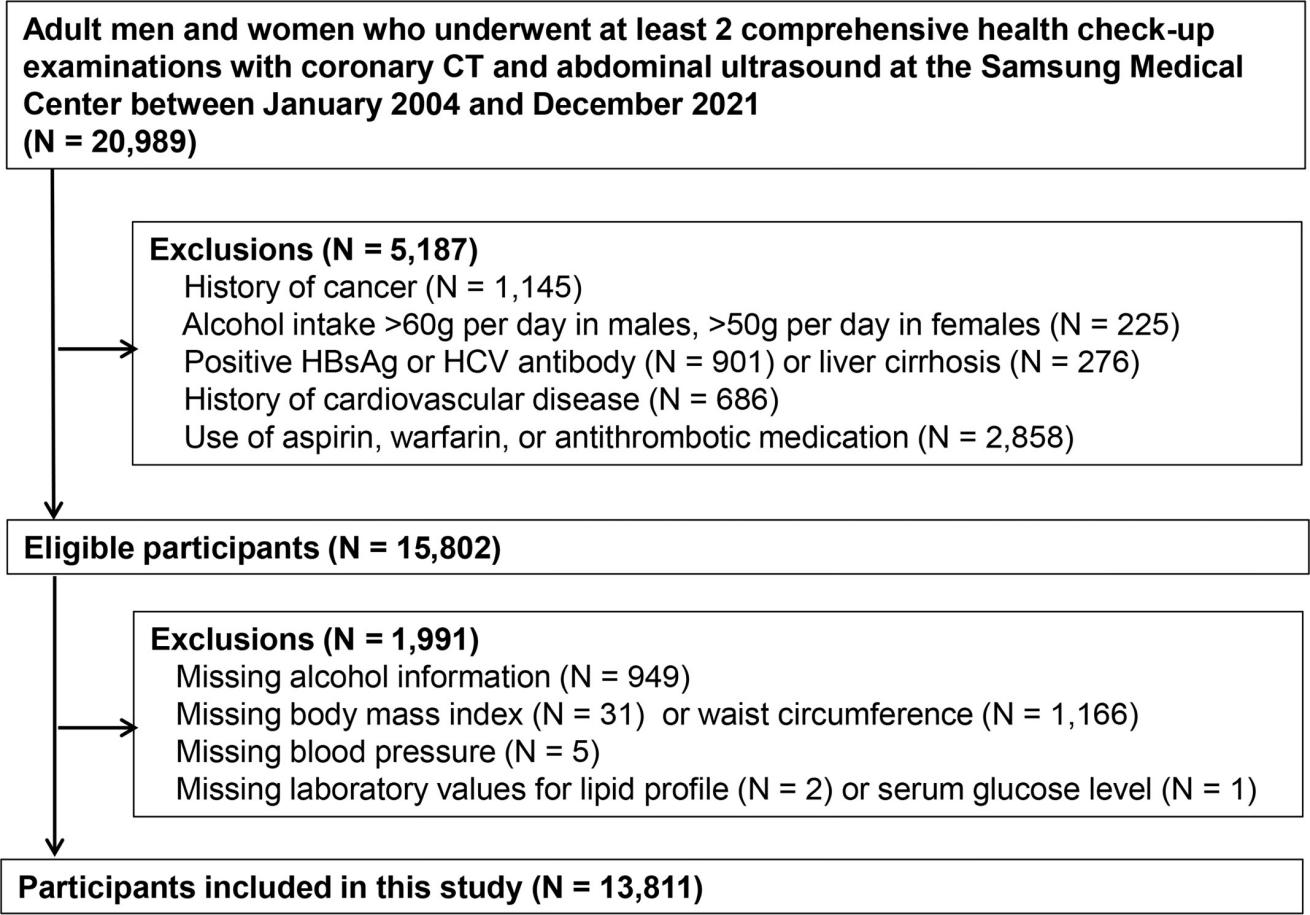
Flow chart of study participants.

We conducted a retrospective cohort analysis of people aged 18 years or older who underwent comprehensive health screening exams at the Samsung Medical Center Health Promotion Center in Seoul, South Korea, from January 1, 2004 to December 31, 2021 (Fig 1). Because our objective was to evaluate the association between SLD and changes in CAC scores, our analysis was restricted to subjects who underwent at least two screening exams that included both a coronary CT scan and an abdominal ultrasound (US) before December 31, 2021 (n = 20,989). We then excluded 5,187 participants who had any of the following conditions: history of cancer (n = 1,145), alcohol intake >60 g per day in males or >50g per day in females (n = 225), positive test for HBsAg or HCV antibody (n = 901), history of liver cirrhosis (n = 276), history of CVD (n = 686), use of aspirin, warfarin, or antithrombotic medication (n = 2,858). Among the eligible participants (n = 15,802), we further excluded 1,991 participants who were missing alcohol information (n = 949), body mass index (BMI) (n = 31), waist circumference (n = 1,166), blood pressure (n = 5), laboratory values for a lipid profile (n = 2) or serum glucose level (n = 1). Because study participants could have more than one exclusion criterion, the final sample size was 13,811. The Institutional Review Board of Samsung Medical Center approved this study (approval no. 2023-08-151) and waived the requirement for informed consent because we used only de-identified data routinely collected during health screening visits. The data were accessed for research purposes from September 1, 2023 to September 30, 2023. The study was conducted in accordance with the Declaration of Helsinki.

### Abdominal US

Abdominal US scans were performed by experienced radiologists unaware of the study aims on LogiQ E9 (GE Healthcare, Milwaukee, WI, USA), iU22 xMatrix (Philips Medical Systems, Cleveland, OH, USA), or ACUSON Sequoia 512 (Siemens, Issaquah, WA, USA) equipment. Images were captured in a standard fashion with the patient in the supine position and the right arm raised above the head. A US diagnosis of hepatic steatosis was made based on standard criteria involving parenchymal brightness, liver-to-kidney contrast, deep beam attenuation, and bright vessel walls [8, 9].

### SLD definition, sub-classifications, and severity

In accordance with the multi-society Delphi consensus statement [2], we diagnosed SLD in participants who showed hepatic steatosis on US. The participants with SLD were categorized as follows: cryptogenic SLD (SLD without metabolic dysfunction or increased alcohol intake), MASLD (SLD with metabolic dysfunction but no increased alcohol intake), and MetALD (SLD with both metabolic dysfunction and increased alcohol intake).

Cardiometabolic dysfunction was also defined in accordance with the multi-society Delphi consensus statement [2] as the presence of one or more of the following risk determinants: 1) BMI ≥ 23 kg/m^2^ or waist circumference > 94 or > 80 cm in males and females, respectively; 2) Fasting glucose levels ≥ 100 mg/dL or hemoglobin A1c ≥ 5.7% or type 2 diabetes or treatment for type 2 diabetes; 3) Blood pressure ≥ 130/85 mmHg or specific antihypertensive drug treatment; 4) Plasma triglycerides ≥ 150 mg/dL or specific lipid lowering treatment; 5) Plasma high-density lipoprotein (HDL) cholesterol ≤ 40 mg/dL for males and ≤ 50 mg/dL for females or specific lipid lowering treatment.

Alcohol intake was assessed using standardized, self-administered questionnaires. Increased alcohol intake was defined as an average daily consumption of 20–50g for females and 30–60g for males (140–350 g/week for females and 210–420 g/week for males).

SLD severity was assessed using the fibrosis-4 (FIB-4) score, which we calculated as age (years) × AST (U/L)/[platelet count (10^9^/L) × ALT (U/L)^1/2^]. We categorized participants as having a low likelihood of liver fibrosis (FIB-4 score < 1.3) and an intermediate or high likelihood of liver fibrosis (≥ 1.3) [10].

### Coronary CT scans

Imaging data for evaluating CAC were acquired using a Brilliance 40 (Philips Medical Systems), VCT LightSpeed 64 (GE Healthcare), or Discovery 750HD (GE Healthcare) multidetector CT scanner. The scans were analyzed on Extended Brilliance Workspace (Philips Medical Systems) or Advantage (GE Healthcare) workstations. CAC scores were calculated as described by Agatston *et al*. [11].

### Other covariates

At each visit, demographic characteristics, smoking status, medical history, and medication use were collected through standardized, self-administered questionnaires. Smoking status was categorized into never or ever smokers. Height, weight, waist circumference, and sitting blood pressure were measured by trained nurses. BMI was calculated as weight in kilograms divided by height in meters squared.

Serum total cholesterol, triglycerides, HDL cholesterol and low-density lipoprotein (LDL) cholesterol were determined using an enzymatic colorimetric method. Serum glucose was measured by the hexokinase/glucose-6-phosphate dehydrogenase method. Aspartate aminotransferase (AST), alanine aminotransferase (ALT), and gamma-glutamyltransferase (GGT) were measured following the International Federation of Clinical Chemistry method. The Department of Laboratory Medicine and Genetics at Samsung Medical Center has participated in several proficiency testing programs operated by the Korean Association of Quality Assurance for Clinical Laboratory, the Asian Network of Clinical Laboratory Standardization and Harmonization, and the College of American Pathologists.

### Statistical analysis

We compared the quantitative progression of CAC scores in participants with and without SLD at baseline using linear mixed models for longitudinal data with random intercepts and random slopes [12]. Because CAC scores are markedly right skewed, the primary analysis used log_e_-transformed scores (CAC + 1) as the outcome and estimated the ratio between the annual progression rates of CAC scores (with 95% confidence intervals [CIs]) of participants with and without SLD at baseline.

We used an adjusted model to account for potential confounding factors, age, sex, and smoking status, at baseline. In an adjusted model to see the association between CAC progression and SLD, metabolic abnormalities (high WC or BMI, high blood pressure, high triglyceride, low HDL-C, and high blood glucose) and increased alcohol intake were additionally adjusted. In an adjusted model to see the association between CAC progression and SLD sub-classifications, metabolic abnormalities and increased alcohol intake were not adjusted because they were already accounted for in the grouping process. For the FIB-4 analyses, the models were not adjusted for age because it is included in calculating the FIB-4 score.

Because participants in our analyses had to have at least 2 screening visits, we used inverse probability weights (IPWs) to correct for potential selection bias in this group. IPWs reweight study participants to give those who are similar to those lost to follow-up after the first coronary CT a higher weight. The IPWs were obtained from a logistic regression model that included all potential participants with at least 1 coronary CT scan and selection criteria similar to those used in this analysis (n = 20,116). All the analyses we report here were corrected using the IPWs (the weighted and unweighted results were very similar).

All reported P values were two-sided, and the significance level was set at 0.05. All analyses were performed using STATA version 13 (StataCorp LP, College Station, TX, USA).

## Results

### Basal characteristics

The mean (SD) age of the study participants was 53.4 (7.2) years, and the prevalence of SLD at baseline was 44.5% (n = 6,152). Compared with participants without SLD, those with SLD were more likely to be male; be smokers; have increased alcohol intake; have elevated serum levels of AST, ALT, and GGT; and be metabolically unhealthy. The median CAC score at baseline was 0 (58.1% participants had a CAC score 0). CAC scores at baseline were higher in participants with SLD than in those without SLD at baseline (median [interquartile range]: 0 [0–26] vs. 0 [0–15], p < 0.001, Table 1). Among the participants with SLD, most had MASLD (89.9%, n = 5,525), followed by MetALD (7.7%, n = 473) and cryptogenic SLD (2.4%, n = 149). Participants with MetALD were most likely to be male; be smokers, have elevated serum levels of AST, ALT, and GGT; and be metabolically unhealthy, and they had the highest CAC scores at baseline among the groups (S1 Table).

**Table 1:** Baseline characteristics of study participants by group (N = 13,811).

Characteristic	No SLD (N = 7,659)	SLD (N = 6,152)	p values
**Age (years)**	53.7 (7.2)	52.9 (7.2)	< 0.01
**Sex, male**	5,870 (76.6)	5,385 (87.5)	< 0.01
**BMI (kg/m** ^**2**^ **)**	23.3 (2.3)	25.6 (2.5)	< 0.01
**Smoking**			< 0.01
Never	3,040 (39.7)	1,773 (28.8)	
Ever	4,484 (58.6)	4,297 (69.9)	
Missing	135 (1.8)	82 (1.3)	
**Increased alcohol intake** ^**a**^	485 (6.3)	478 (7.8)	< 0.01
**AST (U/l)**	23.4 (11.3)	26.6 (12.2)	< 0.01
**ALT (U/l)**	21.9 (19.4)	31.8 (19.7)	< 0.01
**GGT (U/l)**	34.0 (39.6)	48.9 (46.5)	< 0.01
**eGFR (mL/min/1.73 m** ^**2**^ **)**	85.0 (13.0)	85.0 (13.5)	0.99
**Metabolic abnormalities** ^**b**^			
High WC or BMI	4,508 (58.9)	5,412 (88.0)	< 0.01
High blood pressure	2,664 (34.8)	3,014 (49.0)	< 0.01
High triglycerides	1,893 (24.7)	3,236 (52.6)	< 0.01
Low HDL-C	986 (12.9)	1,631 (26.5)	< 0.01
High blood glucose	2,929 (38.2)	3,683 (59.9)	< 0.01
**CAC**	0 (0–15)	0 (0–26)	< 0.01

Values are the mean (SD), median (IQR), or number (%).

ALT, alanine aminotransferase; AST, aspartate aminotransferase; BMI, body mass index; CAC, coronary artery calcium; GGT, gamma-glutamyl transferase; eGFR, estimated glomerular filtration rate; HDL-C, high-density lipoprotein cholesterol; SLD, Steatotic Liver Disease; WC, waist circumference

^a^Increased alcohol intake was defined as average daily 20-50g female, and 30-60g male.

^b^Metabolic abnormalities are defined as follows: BMI ≥ 23 kg/m^2^ or waist circumference > 94 or > 80 cm in males and females, respectively; fasting glucose levels ≥ 100 mg/dL or hemoglobin A1c ≥ 5.7% or type 2 diabetes or treatment for type 2 diabetes; blood pressure ≥ 130/85 mmHg or specific antihypertensive drug treatment; plasma triglycerides ≥ 150 mg/dL or specific lipid lowering treatment; plasma high-density lipoprotein cholesterol ≤ 40 mg/dL for males and ≤ 50 mg/dL for females or specific lipid-lowering treatment.

### Associations between CAC progression and SLD and SLD sub-classifications

The average duration of follow-up was 5.8 years (maximum 17.0 years; average number of visits per participant 3.2). During follow-up, the annual rates of CAC progression (95% CI) in participants with and without SLD at baseline were 18% (17–19%) and 14% (13–14%), respectively (p < 0.01) (Table 2). The multivariable adjusted ratio of progression rates comparing participants with and without SLD was 1.04 (1.03–1.04; p < 0.01) (Table 2).

**Table 2:** Ratio of annual change rates in the coronary artery calcium scores of participants (N = 13,811).

Participant characteristics (or nomenclature)	Rate of CAC progression (95% CI)	Crude ratio of annual CAC progression rates (95% CI)	Adjusted ratio of annual CAC progression rates (95% CI)
**Overall**			
**SLD** ^**a**^			
No SLD	1.14 (1.13, 1.14)	Reference	Reference
SLD	1.18 (1.17, 1.19)	1.04 (1.03, 1.04)	1.04 (1.03, 1.04)
**SLD sub-classifications** ^**b**^			
Cryptogenic SLD	1.11 (1.08, 1.14)	0.97 (0.95, 1.00)	0.98 (0.95, 1.01)
MASLD	1.18 (1.17, 1.18)	1.03 (1.02, 1.04)	1.03 (1.03, 1.04)
MetALD	1.21 (1.19, 1.24)	1.06 (1.04, 1.09)	1.07 (1.04, 1.09)
**Baseline CAC = 0**			
**SLD** ^**a**^			
No SLD	1.11 (1.10, 1.11)	Reference	Reference
SLD	1.15 (1.14, 1.16)	1.04 (1.03, 1.04)	1.04 (1.03, 1.05)
**SLD sub-classifications** ^**b**^			
Cryptogenic SLD	1.08 (1.04, 1.11)	0.98 (0.95, 1.01)	0.98 (0.94, 1.01)
MASLD	1.14 (1.13, 1.15)	1.04 (1.03, 1.04)	1.03 (1.03, 1.04)
MetALD	1.19 (1.16, 1.22)	1.07 (1.04, 1.09)	1.08 (1.04, 1.11)

Values in parentheses are 95% confidence intervals. Annual rates of CAC progression and the ratios of the annual progression rates were estimated from mixed models with random intercepts and random slopes, with log_e_(CAC + 1) as the outcome and inverse probability weighting (see text for details).

CAC, coronary artery calcium; CI, confidence interval; MASLD, metabolic dysfunction–associated steatotic liver disease; MetALD, MASLD with increased alcohol intake; SLD, Steatotic Liver Disease

^a^Adjusted for age, sex, and smoking status (never, ever, or missing), high WC or BMI, high blood pressure, high triglyceride, low HDL-C, high blood glucose, and increased alcohol intake.

^b^Adjusted for age, sex, and smoking status (never, ever or missing)

The adjusted ratios of progression rates comparing participants with cryptogenic SLD, MASLD, and MetALD with those without SLD were 0.98 (95% CI 0.95–1.01; p = 0.14), 1.03 (95% CI 1.03–1.04; p < 0.01), and 1.07 (95% CI 1.04–1.09; p < 0.01), respectively, increasing in the order of cryptogenic SLD, MASLD, and MetALD (Table 2). When only participants with CAC 0 at baseline were studied independently, the associations between SLD and the SLD sub-classifications and CAC development remained similar (Table 2).

### Associations between CAC progression and SLD and its sub-classifications by SLD severity

The progression of CAC scores increased across the categories of SLD severity defined by FIB-4 scores (Table 3). The adjusted ratios of progression rates comparing participants with SLD with FIB-4 <1.3 and SLD with FIB-4 ≥1.3 with those without SLD were 1.03 (95% CI 1.02–1.04; p < 0.01), and 1.05 (95% CI 1.04–1.06; p < 0.01), respectively (Table 3). In MASLD, the CAC score progressed significantly according to the degree of SLD severity, and in MetALD, the CAC score increased according to the degree of SLD severity, but this did not reach statistical significance.

**Table 3:** Ratio of annual change rates in the coronary artery calcium scores by SLD severity.

Participant characteristics (or nomenclature)	Rate of CAC progression (95% CI)	Crude ratio of annual CAC progression rates (95% CI)	Adjusted ratio of annual CAC progression rates (95% CI)
**SLD severity** ^**a**^			
No SLD	1.14 (1.13, 1.14)	Reference	Reference
SLD with FIB-4 < 1.3	1.17 (1.17, 1.18)	1.03 (1.02, 1.04)	1.03 (1.02, 1.04)
SLD with FIB-4 ≥ 1.3	1.20 (1.19, 1.21)	1.05 (1.04, 1.06)	1.05 (1.04, 1.06)
**SLD sub-classifications severity** ^**b**^			
Cryptogenic SLD with FIB-4 < 1.3	1.09 (1.06, 1.12)	0.96 (0.93, 0.99)	0.96 (0.93, 1.00)
Cryptogenic SLD with FIB-4 ≥ 1.3	1.14 (1.09, 1.20)	1.00 (0.95, 1.06)	1.01 (0.95, 1.07)
MASLD with FIB-4 < 1.3	1.17 (1.17, 1.18)	1.03 (1.02, 1.04)	1.03 (1.02, 1.04)
MASLD with FIB-4 ≥ 1.3	1.20 (1.18, 1.21)	1.05 (1.04, 1.06)	1.05 (1.04, 1.06)
MetALD with FIB-4 < 1.3	1.20 (1.18, 1.23)	1.06 (1.03, 1.08)	1.05 (1.03, 1.08)
MetALD with FIB-4 ≥ 1.3	1.22 (1.18, 1.27)	1.07 (1.04, 1.11)	1.09 (1.05, 1.12)

Values in parentheses are 95% confidence intervals. Annual rates of CAC progression and ratios of annual progression rates were estimated from mixed models with random intercepts and random slopes, with log_e_(CAC + 1) as the outcome and inverse probability weighting (see text for details).

Abbreviations: CAC, coronary artery calcium; CI, confidence interval; FIB-4, fibrosis-4; MASLD, metabolic dysfunction–associated steatotic liver disease; MetALD, MASLD with increased alcohol intake; SLD, Steatotic Liver Disease.

^a^Adjusted for sex, and smoking status (never, ever, or missing), high WC or BMI, high blood pressure, high triglyceride, low HDL-C, high blood glucose, and increased alcohol intake.

^b^Adjusted for sex, and smoking status (never, ever or missing)

## Discussion

In this large longitudinal study, we found that participants with SLD had a higher CVD risk, indicated as faster progression of their CAC scores, than participants without SLD. The association persisted after adjusting for traditional risk factors, metabolic abnormalities and increased alcohol intake. When participants with SLD were further categorized into the newly proposed SLD sub-classifications, CAC progression increased significantly in the order of cryptogenic SLD, MASLD, and MetALD. SLD severity and the progression of CAC scores correlated positively across SLD and two of the SLD sub-classifications (MASLD and MetALD).

Our group has previously reported an association between NAFLD and CAC progression, demonstrating a faster CAC progression rate for participants with NAFLD in an analysis of 4,731 adults who received screening exams between 2004 and 2013 [13]. Several other studies have also shown that NAFLD increases the risk of CAC progression [13–15]. Compared with our previous work, the enrollment period in this study was extended (between 2004 and 2021), and participants with increased alcohol intake (30–60 g/day in males, 20–50 g/day in females) were additionally enrolled because the new SLD definition includes participants with increased alcohol intake. Under the new sub-classifications system, individuals with traditional NAFLD are divided into cryptogenic SLD and MASLD. In this study, the CAC progression risk was higher among those with MASLD than cryptogenic SLD. Compared with cryptogenic SLD, MASLD requires the presence of metabolic dysfunction [2]. The key pathophysiological pathways linking NAFLD and coronary atherosclerosis are supposed to be insulin resistance [16], subclinical inflammation [16], endothelial dysfunction [17], and changed lipid profiles (atherogenic dyslipidemia) [18]. When NAFLD and hypertension coexist, early vascular alterations resulting in vascular damage have been reported [19], and the level of inflammation and insulin resistance were higher when diabetes coexisted with hepatic steatosis [20]. Thus, it is not surprising to see that an increased risk of CAC progression is evident in participants with MASLD, who have metabolic dysfunction, but not in those with cryptogenic SLD. In studies comparing the risk of CVD between metabolic dysfunction-associated fatty liver disease (MAFLD) and NAFLD, MAFLD predicted CVD risk better than NAFLD [21, 22]. That can be also explained by the presence of metabolic dysfunction in the definition of MAFLD. These findings all imply that the increased risk of CAC progression in participants with MASLD compared to those with cryptogenic SLD is associated with metabolic dysfunction. Also, in terms of stratifying the CAC risk, categorizing individuals with SLD as having cryptogenic SLD or MASLD is clinically relevant.

In the new sub-classifications system, individuals with hepatic steatosis and increased alcohol intake (30–60 g/day in males, 20–50 g/day in females) are diagnosed with MetALD. This study found that participants with MetALD were at the highest risk of CAC progression, suggesting that a certain level of alcohol intake (30–60 g/day in males, 20–50 g/day in females) could have harmful effects on coronary atherosclerosis in participants with SLD. The association between alcohol intake and coronary atherosclerosis is complex and requires careful interpretation. Low-to-moderate alcohol use has been demonstrated to lower the risk of CVD and atherosclerosis [23, 24]. The reduced risk is probably related to alcohol’s favorable pleiotropic effects on lipids, adhesion molecules, platelet activation, and oxidative stress [25]. Chronic high-dose consumption of alcohol, on the other hand, leads to cardiovascular diseases and atherosclerosis advancement. This appears to be due to the metabolism of alcohol, which leads to the formation of acetaldehyde that is oxidized to acetate and leads to the generation of reactive oxygen species that have a toxic effect on the formation of atherosclerotic plaques [26]. Of note, the same amount of alcohol intake can have different effects depending on the health of each individual [27]. In addition, alcohol can have different effects on human health depending not only on the amount, but also on the type and pattern of alcohol consumption [26, 28]. The exact mechanism linking MetALD and CAC progression is unclear and thus requires further study.

In this study, we also assessed whether risk of CAC progression differs by SLD severity, which we defined with the FIB-4 index. SLD severity and CAC progression correlated positively across SLD and its sub-classifications. Liver fibrosis has been proposed as an independent risk factor for subclinical atherosclerosis, possibly due to its role in subclinical inflammation and oxidative stress [17]. In a meta-analysis of 12 studies of NAFLD patients, the OR for the association between liver fibrosis and subclinical atherosclerosis was 2.18 (95% CI 1.62–2.93), and in the subgroup analysis, the OR for the association with CAC was 2.76 (95% CI 1.18–6.45) [29]. Those findings are in line with our finding that SLD patients with more severe liver fibrosis had more rapid advances in coronary atherosclerosis.

This study has some limitations. Because we used a health screening cohort, selection bias could exist. The health screening cohort usually consists of individuals who are interested in their health, so our results might not be generalizable to the general population. The gold standard for diagnosing the presence of hepatic steatosis is a liver biopsy. In this study, we used US, a practical and safe method widely used to assess hepatic steatosis, but classification bias might nonetheless exist because measurement errors have been reported when using US to assess the presence of hepatic steatosis [30]. Alcohol consumption was evaluated through self-administered structured questionnaires. Self-reported amounts of alcohol consumption might not accurately reflect the actual amount of alcohol intake [31]. Residual confounding that could explain the associations observed in this study might also exist.

In summary, SLD was independently associated with the progression of coronary atherosclerosis. The risk of CAC progression differed by SLD sub-classifications, and those with MetALD had the highest risk. Participants with SLD with advanced fibrosis were at higher risk of CAC progression than those with SLD without advanced fibrosis. The newly proposed SLD and its sub-classifications system can well differentiate CVD risk, suggesting that assessing and stratifying SLD participants according to the presence of metabolic dysfunction and the amount of alcohol consumption is clinically relevant.

## Supporting information

S1 Table Baseline characteristics of study participants by group (N = 13,806). (DOCX)

## List of abbreviations in the order of appearance

SLD: Steatotic Liver Disease
CAC: coronary artery calcium
MASLD: metabolic dysfunction–associated steatotic liver disease
MetALD: MASLD with increased alcohol intake
FIB-4: fibrosis-4
NAFLD: nonalcoholic fatty liver disease
CVD: cardiovascular disease
US: ultrasound
BMI: body mass index
HDL: high-density lipoprotein
LDL: low-density lipoprotein
AST: aspartate aminotransferase
ALT: alanine aminotransferase
GGT: gamma-glutamyltransferase
CI: confidence interval
IPWs: inverse probability weights
eGFR: estimated glomerular filtration rate
WC: waist circumference
