# Supplementary material for: Steatotic liver disease and its newly proposed sub-classifications correlate with progression of the coronary artery calcium score
Source: PLoS One. 2024 Mar 26;19(3):e0301126. doi: 10.1371/journal.pone.0301126 (PMC10965078; doi:10.1371/journal.pone.0301126)
Supplement: S1 Table — (DOCX) [file pone.0301126.s001.docx]

**S1 Table. Baseline characteristics of study participants by group (N = 13,806).**

| **Characteristic** | **No SLD (N = 7,659)** | **Cryptogenic SLD (N = 149)** | **MASLD (N = 5,525)** | **MetALD (N = 473)** | **p for trends** |
| --- | --- | --- | --- | --- | --- |
| **Age (years)** | 53.7 (7.2) | 51.1 (6.1) | 53.1 (7.3) | 51.3 (6.1) | < 0.01 |
| **Sex, male** | 5,870 (76.6) | 124 (83.2) | 4,788 (86.7) | 468 (98.9) | < 0.01 |
| **BMI (kg/m^2^)** | 23.3 (2.3) | 21.8 (1.0) | 25.6 (2.4) | 26.3 (2.5) | < 0.01 |
| **Smoking** |  |  |  |  | < 0.01 |
| Never | 3,040 (39.7) | 60 (40.3) | 1,659 (30.0) | 54 (11.4) |  |
| Ever | 4,484 (58.6) | 88 (59.1) | 3,789 (68.6) | 415 (87.7) |  |
| Missing | 135 (1.8) | 1 (0.7) | 77 (1.4) | 4 (0.9) |  |
| **Increased alcohol intake^a^** | 485 (6.3) | 0 | 0 | 473 (100) | < 0.01 |
| **AST (U/l)** | 23.4 (11.3) | 22.8 (8.9) | 26.3 (11.6) | 31.0 (17.5) | < 0.01 |
| **ALT (U/l)** | 21.9 (19.4) | 22.4 (10.8) | 31.8 (19.6) | 34.7 (22.2) | < 0.01 |
| **GGT (U/l)** | 34.0 (39.6) | 30.4 (21.0) | 46.1 (39.3) | 87.7 (90.5) | < 0.01 |
| **eGFR (mL/min/1.73 m^2^)** | 85.0 (13.0) | 86.5 (11.8) | 84.6 (13.4) | 88.4 (13.9) | 0.11 |
| **Metabolic abnormalities^b^** |  |  |  |  |  |
| High WC or BMI | 4,508 (58.9) | 0 | 4,909 (89.9) | 443 (93.8) | < 0.01 |
| High blood pressure | 2,664 (34.8) | 0 | 2,746 (49.7) | 268 (56.7) | < 0.01 |
| High triglycerides | 1,893 (24.7) | 0 | 2,948 (53.4) | 288 (60.9) | < 0.01 |
| Low HDL-C | 986 (12.9) | 0 | 1,526 (27.6) | 105 (22.2) | < 0.01 |
| High blood glucose | 2,929 (38.2) | 0 | 3,346 (60.6) | 337 (71.3) | < 0.01 |
| **CAC** | 0 (0-15) | 0 (0-3) | 0 (0-26) | 0 (0-39) | < 0.01 |

Values are the mean (SD), median (IQR), or number (%).

ALT, alanine aminotransferase; AST, aspartate aminotransferase; BMI, body mass index; CAC, coronary artery calcium; GGT, gamma-glutamyl transferase; eGFR, estimated glomerular filtration rate; HDL-C, high-density lipoprotein cholesterol; MASLD, metabolic dysfunction–associated steatotic liver disease; MetALD, MASLD with increased alcohol intake; SLD, Steatotic Liver Disease; WC, waist circumference.

^a^Increased alcohol intake was defined as average daily 20-50g female, and 30-60g male.

^b^Metabolic abnormalities are defined as follows: BMI ≥ 23 or waist circumference > 94 or > 80 cm in males and females, respectively; fasting glucose levels ≥ 100 mg/dL or hemoglobin A1c ≥ 5.7% or type 2 diabetes or treatment for type 2 diabetes; blood pressure ≥ 130/85 mmHg or specific antihypertensive drug treatment; plasma triglycerides ≥ 150 mg/dL or specific lipid lowering treatment; plasma high-density lipoprotein cholesterol ≤ 40 mg/dL for males and ≤ 50 mg/dL for females or specific lipid lowering treatment.
